# *Ehrlichia chaffeensis* TRP75 Interacts with Host Cell Targets Involved in Homeostasis, Cytoskeleton Organization, and Apoptosis Regulation To Promote Infection

**DOI:** 10.1128/mSphere.00147-18

**Published:** 2018-04-11

**Authors:** Tian Luo, Shubhajit Mitra, Jere W. McBride

**Affiliations:** a Department of Pathology, University of Texas Medical Branch, Galveston, Texas, USA; b Department of Microbiology and Immunology, University of Texas Medical Branch, Galveston, Texas, USA; c Center for Biodefense and Emerging Infectious Diseases, University of Texas Medical Branch, Galveston, Texas, USA; d Sealy Center for Vaccine Development, University of Texas Medical Branch, Galveston, Texas, USA; e Institute for Human Infections and Immunity, University of Texas Medical Branch, Galveston, Texas, USA; University of Kentucky

**Keywords:** *Ehrlichia chaffeensis*, apoptosis, cytoskeleton organization, effector-host interaction, homeostasis, tandem repeat protein

## Abstract

Ehrlichia chaffeensis is an obligately intracellular bacterium that exhibits tropism for mononuclear phagocytes. The mechanisms involved in E. chaffeensis infection of the host cell and evasion of host defenses are not fully defined, but a subset of type 1 secreted tandem repeat protein (TRP) effectors play important roles. Recently, we determined molecular interactions of TRP120, TRP47, and TRP32 with the eukaryotic host cell. In this investigation, we used yeast two-hybrid analysis to reveal that another E. chaffeensis tandem repeat protein, TRP75, interacts with a diverse group of human proteins associated with organismal and tissue homeostasis, multiple metabolic processes and regulation, response to reactive oxygen species, signal transduction, and protein modifications. Thirteen identified host target proteins associated with actin cytoskeleton reorganization or apoptosis were examined in detail and confirmed to interact with TRP75 at different levels as determined by coimmunoprecipitation assays. These protein interactions were visualized by immunofluorescence confocal microscopy during infection and colocalized with *Ehrlichia* morulae with different intensities. Moreover, small interfering RNAs (siRNAs) (*n* = 86) were used to knock down identified TRP75-interacting host proteins separately, and their influence on ehrlichial infection was investigated by real-time quantitative PCR (qPCR). Knockdown of 74/86 (86%) TRP75 target proteins had a significant negative effect on ehrlichial infection. The results of this study further support the idea of a role of *Ehrlichia* TRPs as effectors that interact with a complex array of host proteins to promote ehrlichial infection.

**IMPORTANCE** Human monocytic ehrlichiosis (HME) is caused by an obligatory intracellular bacterium, E. chaffeensis, and is one of the most prevalent, life-threatening emerging infectious zoonoses in the United States. The mechanisms through which E. chaffeensis invades and establishes an intracellular niche are not well understood but are dependent on secreted ehrlichial effector proteins. The significance of this study is in addressing how intracellular pathogens, particularly those with small genomes such as *Ehrlichia*, exploit a limited number of secreted effector proteins such as tandem repeat proteins (TRPs) to manipulate complex eukaryotes and to regulate host cell processes through molecular pathogen-host interplay. The results of our studies highlight the broader role of ehrlichial TRPs in promoting infection and help define the mechanisms through which obligately intracellular bacteria modulate host cell function for survival.

## Abstract (precis)

Human monocytic ehrlichiosis (HME) is caused by an obligatory intracellular bacterium, E. chaffeensis, and is one of the most prevalent, life-threatening emerging infectious zoonoses in the United States. The mechanisms through which E. chaffeensis invades and establishes an intracellular niche are not well understood but are dependent on secreted ehrlichial effector proteins. The significance of this study is in addressing how intracellular pathogens, particularly those with small genomes such as *Ehrlichia*, exploit a limited number of secreted effector proteins such as tandem repeat proteins (TRPs) to manipulate complex eukaryotes and to regulate host cell processes through molecular pathogen-host interplay. The results of our studies highlight the broader role of ehrlichial TRPs in promoting infection and help define the mechanisms through which obligately intracellular bacteria modulate host cell function for survival.

## INTRODUCTION

Ehrlichia chaffeensis is an obligately intracellular bacterium that causes human monocytotropic ehrlichiosis (HME), an emerging human zoonosis (1). *Ehrlichia* bacteria have a limited number of genes but are able to infect and replicate within membrane-bound cytoplasmic vacuoles in mononuclear phagocytes by evading innate and adaptive host defense mechanisms (1, 2). During infection, *Ehrlichia* bacteria reprogram numerous host cell processes, including gene expression, signal transduction, cell cycle and differentiation, immune responses, membrane trafficking, and apoptosis; however, the mechanism and the effector proteins involved remain largely undetermined (3, 4).

E. chaffeensis has a small group of tandem repeat proteins (TRPs), including TRP32, TRP47, TRP75, and TRP120, which elicit strong antibody responses that are protective (5–9). TRP47 and TRP120 are exclusively detected on dense-cored ehrlichiae only, while TRP32 and TRP75 are constitutively expressed on both dense-cored and reticulate ehrlichiae (5, 7, 9, 10). TRP75 is lysine rich and slightly acidic (pI, ~5.5) while TRP32, TRP47, and TRP120 are serine rich and highly acidic (pI, 4.1 to 4.2) (5–7, 9). The native TRP75 is tyrosine phosphorylated, although the specific modified residues and tyrosine kinases involved remain undefined (9). Unlike the other three TRPs, TRP75 has a lipobox sequence in the N-terminal region and thus is predicted to be a lipoprotein (11). In recent years, E. chaffeensis TRPs (TRP32, TRP47, and TRP120) have been defined as type 1 secretion system substrates and effectors that are involved in complex molecular strategies to modulate host cellular processes (12–15). Molecular interactions between *Ehrlichia* TRPs and host cells involve an array of host proteins associated with major biological processes, including cell signaling, transcription and translation, metabolism, protein trafficking, and apoptosis (12, 13, 15). Furthermore, TRP32 and TRP120 are nucleomodulins that directly bind a specific DNA motif in host genes associated with differentiation and proliferation, signal transduction, transcriptional regulation, and apoptosis (16, 17). However, the molecular interactions between TRP75 and host cell proteins have not been previously investigated.

In order to define the function and role of TRP75 in ehrlichial pathology and to further understand the molecular interactions between *Ehrlichia* TRPs and host cells, a yeast two-hybrid (Y2H) assay was used to determine whether, consistent with other TRPs, E. chaffeensis TRP75 interacts with a large group of host proteins involved in various cellular processes. Knockdown of most TRP75 host targets had a negative effect on ehrlichial infection. Therefore, the results of this study demonstrate that TRP75 is also an important effector protein involved in molecular interactions with the host cell to promote infection.

## RESULTS

### Analysis of E. chaffeensis TRP75 interactions with human proteins by yeast two-hybrid (Y2H) assays.

**FIG 1:**
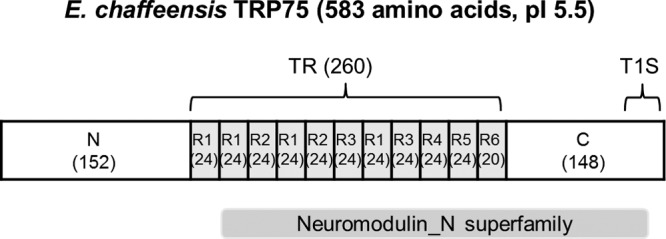
E. chaffeensis TRP75 regions and conserved domains. (Top) Schematic of E. chaffeensis TRP75 showing the regions of N termini, TRs, and C termini (numbers of amino acids are indicated in parentheses; R1 to R6, 6 variations of repeats). The TR region is shown in gray. The last repeat R6 is incomplete (20 amino acids). T1S, type 1 secretion signal. (Bottom) A putative conserved domain (neuromodulin_N superfamily) was identified using the NCBI Conserved Domain Database.

E. chaffeensis TRP75 (ECH_0558) is a 583-amino acid protein containing 10.5 nearly identical 24-mer tandem repeats (TRs) flanked by N (152 amino acids) and C (171 amino acids) termini (Fig. 1). Similarly to other E. chaffeensis TRPs, the region of last 50 C-terminal residues of TRP75 is LDAVTSIF rich (46%) and KHPMWC poor (14%), suggesting that TRP75 is also a type 1 secretion system substrate (14). A conserved protein domain of the neuromodulin_N superfamily (E value = 8.97e−04) has been found in the TRP75 TR and C-terminal regions (amino acids 174 to 562) by BLASTP (protein-protein BLAST) analysis (http://blast.ncbi.nlm.nih.gov/Blast.cgi) (Fig. 1). The significance of this domain in TRP75 needs to be further confirmed and identified.

E. chaffeensis TRP75 was predicted to contain a transmembrane helix (amino acids 1 to 22) using TMHMM analysis (http://www.cbs.dtu.dk/services/TMHMM). Thus, the TRP75 fragment (amino acids 23 to 583) that did not contain this N-terminal sequence was used for yeast two-hybrid screening. In total, 58.4 million clones of a human macrophage cDNA library (~5.8-fold the complexity of the library) were screened, and 243 His-positive (His^+^) colonies were obtained on a selective medium. The prey fragments of the positive clones were amplified and sequenced, and the sequences were analyzed using the GenBank database.

**FIG 2:**
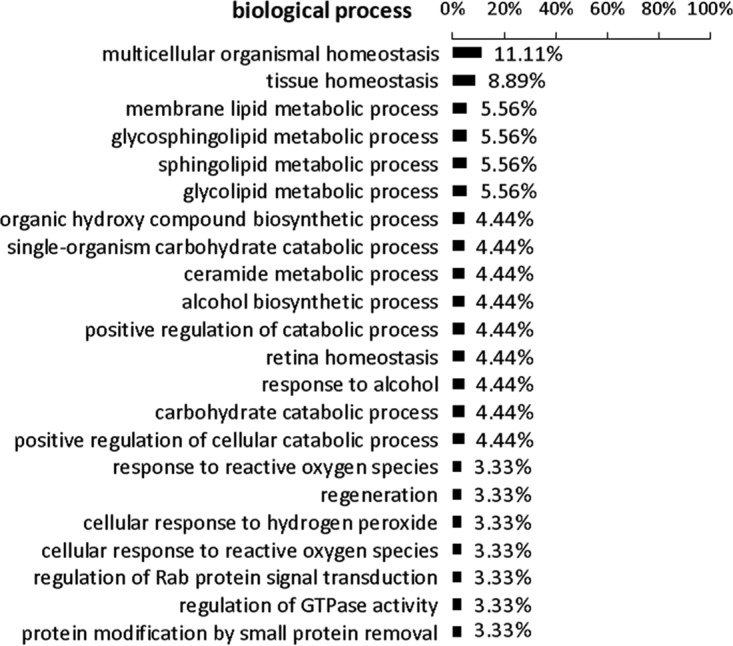
Gene ontology analysis of TRP75-interacting proteins identified by Y2H on biological processes.

After sequence analysis, 90 different interacting human proteins were identified (see Table S1 in the supplemental material). The Babelomics FatiGO tool (version 5; http://babelomics.bioinfo.cipf.es) was used to perform a functional ontology analysis of the 90 identified TRP75-interacting targets (18, 19). Proteins were classified in the human gene ontology database of biological processes that may be affected by TRP75, and results of a FatiGO single enrichment analysis performed for the rest of the human genome (*P* < 0.05) indicated that most of the target proteins were involved in major biological processes, including multicellular organismal and tissue homeostasis (*P* < 1e−8); multiple metabolic processes, including those involving lipids and carbohydrates; regulation of catabolic processes; organic biosynthetic processes; responses to reactive oxygen species; signal transduction; and protein modification (Fig. 2). Homeostasis is the stable condition of an organism and of its internal environment. It is maintained by many regulatory mechanisms and is dependent on many variables, and many diseases result from homeostatic failure (20). The results of ontology analyses of identified TRP75-interacting targets indicated that one primary function is to influence host cell homeostasis.

10.1128/mSphere.00147-18.4 TABLE S1  A list of 90 human proteins that interact with E. chaffeensis TRP75 determined by yeast two-hybrid assay. Download TABLE S1, PDF file, 0.1 MB. Copyright © 2018 Luo et al. 2018 Luo et al. This content is distributed under the terms of the Creative Commons Attribution 4.0 International license.

### Confirmation of interactions by coimmunoprecipitation (Co-IP).

In order to fully explore the function of all TRP75 targets, we further examined the annotations using general gene information associated with the NCBI gene entry (https://www.ncbi.nlm.nih.gov/gene) and a literature review of all of the targets, and, interestingly, we found that many of the targets are associated with actin cytoskeleton reorganization and apoptosis. Moreover, we also found that some targets were enriched with respect to the actin cytoskeleton by the use of GO-Slim cellular component analysis of the PANTHER classification system (http://pantherdb.org/). Thus, to confirm the interactions between E. chaffeensis TRP75 and human proteins identified by Y2H, 13 candidate proteins from the cell processes that appeared to be those most highly targeted by *Ehrlichia* TRP75-host interactions were selected from the 90 interacting proteins, including SLC4A7 (solute carrier family 4 member 7), ARPC5 (actin-related protein 2/3 complex subunit 5), LCP1 (lymphocyte cytosolic protein 1 [L-plastin]), PLEK (pleckstrin), TPM4 (tropomyosin 4), EEF1A1 (eukaryotic translation elongation factor 1 alpha 1), ITGB2 (integrin subunit beta 2), PRDX3 (peroxiredoxin 3), PRKAA1 (protein kinase AMP-activated catalytic subunit alpha 1), PSMC5 (proteasome 26S subunit, ATPase 5), RB1CC1 (RB1-inducible coiled-coil 1), SEPW1 (selenoprotein W, 1), and STAT3 (signal transducer and activator of transcription 3) (Table 1). All of these proteins are associated with actin cytoskeleton reorganization or apoptosis of the host cell, except for SLC4A7, which is involved in regulation of intracellular pH and host cell homeostasis.

**TABLE 1:** Summary of 13 human proteins that interact with E. chaffeensis TRP75 determined by yeast two-hybrid assay

Category	Gene symbol	Full name	Annotation
Solute carrier	SLC4A7	Solute carrier family 4 member 7	Sodium bicarbonate cotransporter; regulation of intracellular pH
			
Actin binding or actin related	ARPC5	Actin-related protein 2/3 complex subunit 5	Actin nucleation; actin cytoskeleton organization; movement of cell or subcellular component; phagocytosis; cell signaling pathway
	LCP1	Lymphocyte cytosolic protein 1 (l-plastin)	Actin-binding protein involved in actin filament bundle assembly; extracellular matrix disassembly; cell migration; intracellular protein transport.
	PLEK	Pleckstrin	Actin cytoskeleton reorganization; vesicle docking involved in exocytosis; signaling
	TPM4	Tropomyosin 4	Actin-binding proteins involved in the cytoskeleton reorganization
			
Apoptosis related	EEF1A1	Eukaryotic translation elongation factor 1 alpha 1	Protein translation; regulation of chaperone-mediated autophagy; regulation of transcription; apoptosis
	ITGB2	Integrin subunit beta 2	Positive regulation of apoptosis; cell signaling
	PRDX3	Peroxiredoxin 3	A mitochondrial antioxidant; negative regulation of apoptosis; cellular proliferation and differentiation
	PRKAA1	Protein kinase AMP-activated catalytic subunit alpha 1	Regulation of apoptosis; cell signaling; regulation of transcription; vesicle-mediated transport
	PSMC5	Proteasome 26S subunit, ATPase 5	Negative regulation of apoptosis; cell signaling; ubiquitin- dependent protein catabolic process
	RB1CC1	RB1-inducible coiled-coil 1	Negative regulation of apoptosis; cell signaling
	SEPW1	Selenoprotein W, 1	Antioxidant; cell signaling
	STAT3	Signal transducer and activator of transcription 3	Transcription activator; regulation of apoptosis; cell signaling

**FIG 3:**
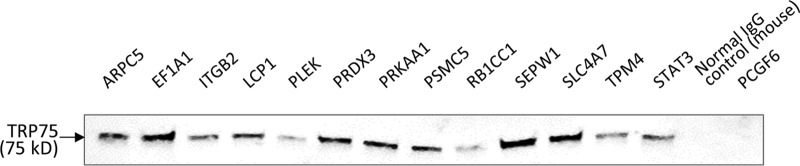
Coimmunoprecipitation and chemiluminescence detection of TRP75 from E. chaffeensis-infected THP-1 cells. Co-IP was performed using specific antibody against selected TRP75-interacting target protein, followed by chemiluminescence detection of TRP75. A normal mouse IgG and an antibody against PCGF6 (polycomb group ring finger protein 6, a TRP120-interacting protein) were used as negative controls. Data are representative of results from *n* = 3 experiments.

Co-IP data indicated that all 13 target antibodies precipitated TRP75, and the results were detectable by chemiluminescent Western blotting at different levels of intensity. Two negative-control antibodies, a normal mouse IgG and an antibody against PCGF6 (polycomb group ring finger protein 6, a TRP120-interacting protein), did not precipitate TRP75 at a detectable level (Fig. 3; see also Fig. S2 in the supplemental material [full blot]).

### TRP75 colocalizes with human target proteins in E. chaffeensis-infected THP-1 cells.

**FIG 4:**
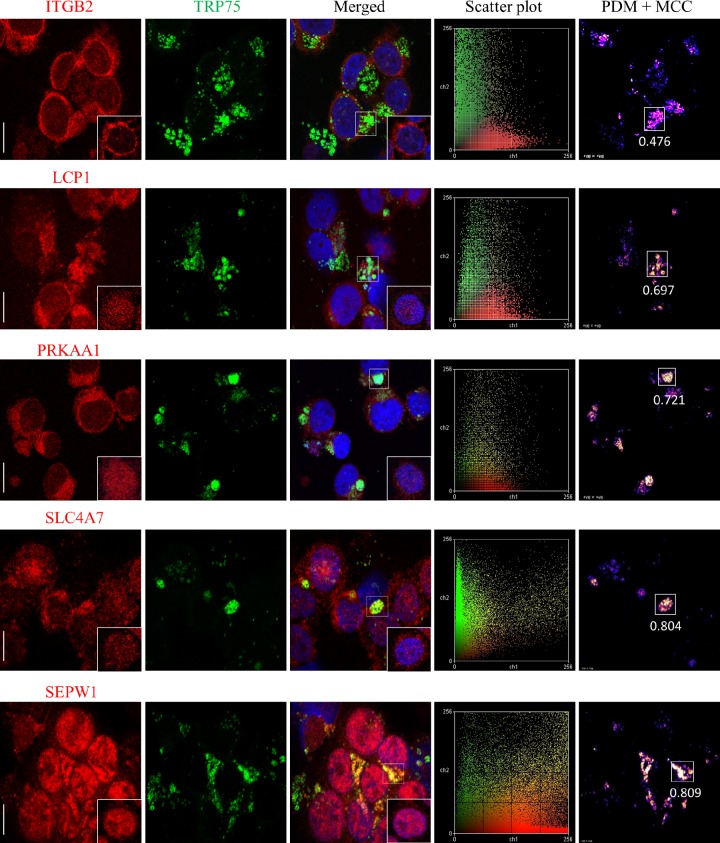
Colocalization of TRP75 with interacting host proteins in E. chaffeensis-infected THP-1 cells. Fluorescent confocal microscopy of infected (3 days p.i.) THP-1 cells stained with host protein antibody (red), TRP75 antibody (green), and 4,6′-diamidino-2-phenylindole (blue, showing the nucleus) was performed. Merged pictures show colocalization of E. chaffeensis TRP75-labeled morulae with host protein. The insets show the distribution of host proteins in uninfected THP-1 cells. Bars, 10 µm. Intensity scatter plots exhibit the distribution of green and red pixels for the represented images. Positive PDM images demonstrate different colocalization points for the represented images. The Manders colocalization coefficient (MCC) is represented on each PDM image next to the calculated region of interest (rectangle) demonstrating the strength of colocalization based on the intensity correlation.

**FIG 5:**
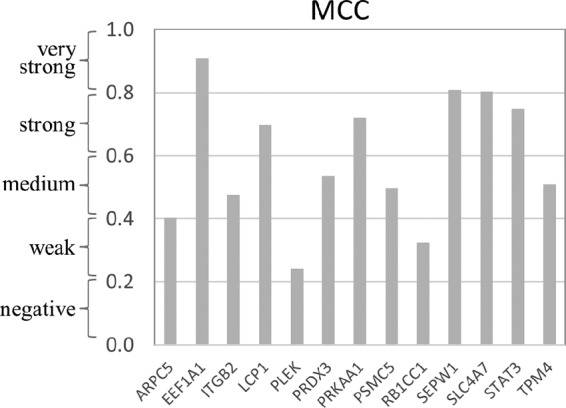
Manders colocalization coefficient (MCC) values quantify the different levels of colocalization between TRP75 and interacting host proteins in E. chaffeensis-infected THP-1 cells. >0.8, very strong colocalization; ~0.60 to ~0.8, strong colocalization; ~0.4 to ~0.6, moderate colocalization; ~0.2 to ~0.4, weak colocalization; ~0 to ~0.2, negative colocalization.

The merged confocal laser images of doubly immunofluorescence-labeled E. chaffeensis-infected THP-1 cells indicated that all 13 of selected host proteins (red) mentioned above colocalized with E. chaffeensis morulae that stained with anti-TRP75 antibody (green), consistent with our Co-IP results, but that they also exhibited very different levels of colocalization strength (Fig. 4; see also Fig. S1 [left 3 columns of images]). Intensity correlation analysis demonstrated a positive PDM (i.e., product of the differences from the mean) value, which correlated with strong or weak colocalization of TRP75 and target proteins in the region. To further confirm the colocalization of host proteins with TRP75-expressing morulae, intensity scatter and Manders colocalization coefficient (MCC) data in the colocalized regions were analyzed (Fig. 4; see also Fig. S1 [right 2 columns of images]). An intensity scatter plot of the selected regions demonstrated strong or weak fluorescence intensity for both the red and green channels, which was consistent with the PDM and MCC results. On the basis of the MCC values, TRP75 exhibited very strong colocalization (MCC, >0.8) with target proteins EEF1A1, SEPW1, and SLC4A7; strong colocalization (MCC, ~0.6 to ~0.8) with LCP1, PRKAA1, and STAT3; a medium level of colocalization (MCC, ~0.4 to ~0.6) with ARPC5, ITGB2, PRDX3, PSMC5, and TPM4; and weak colocalization (MCC, ~0.2 to ~0.4) with PLEK and RB1CC1 (Fig. 5). Compared to uninfected cells, redistribution of some TRP75 target proteins in E. chaffeensis-infected cells was observed. For example, LCP1, PRKAA1, and SLC4A7, which interact with TRP75 strongly, were mostly associated with E. chaffeensis morulae (Fig. 4), while in uninfected THP-1 cells, they exhibited diffuse or punctate distribution throughout the cell (Fig. 4 insets; see also Fig. S3). The anti-TRP75 antibody did not stain uninfected cells (Fig. S3).

10.1128/mSphere.00147-18.1 FIG S1  Colocalization of TRP75 with interacting host proteins in E. chaffeensis-infected THP-1 cells. Fluorescent confocal microscopy of infected (3 days p.i.) THP-1 cells stained with host protein antibody (red), TRP75 antibody (green), and 4,6′-diamidino-2-phenylindole (blue, showing the nucleus) was performed. Merged pictures show colocalization of E. chaffeensis TRP75-labeled morulae with host protein. The boxed insets show the distribution of host proteins in uninfected THP-1 cells. Bars, 10 µm. Intensity scatter plots exhibit the distribution of green and red pixels for the represented images. Positive PDM images demonstrate different colocalization points for the represented images. The Manders colocalization coefficient (MCC) is represented on each PDM image next to the calculated region of interest (rectangle) demonstrating the strength of colocalization based on the intensity correlation. Download FIG S1, PDF file, 1 MB. Copyright © 2018 Luo et al. 2018 Luo et al. This content is distributed under the terms of the Creative Commons Attribution 4.0 International license.

10.1128/mSphere.00147-18.2 FIG S2  Coimmunoprecipitation and chemiluminescence detection of TRP75 from E. chaffeensis-infected THP-1 cells (full blot). The top bands represent the full-size TRP75, and the bottom bands represent the fragment of TRP75 processed through undefined proteolytic activity. Co-IP was performed using specific antibody against selected TRP75-interacting target protein, followed by chemiluminescence detection of TRP75. A normal-mouse IgG and an antibody against PCGF6 (polycomb group ring finger protein 6, a TRP120-interacting protein) were used as negative controls. Data are representative of results from *n* = 3 experiments. Download FIG S2, PDF file, 0.3 MB. Copyright © 2018 Luo et al. 2018 Luo et al. This content is distributed under the terms of the Creative Commons Attribution 4.0 International license.

10.1128/mSphere.00147-18.3 FIG S3  E. chaffeensis TRP75 target proteins in uninfected THP-1 cells. Fluorescent confocal microscopy of uninfected THP-1 cells stained with host protein antibody (red), TRP75 antibody (green), and 4,6′-diamidino-2-phenylindole (blue, showing the nucleus) was performed. Merged pictures show the distribution of TRP75 target proteins in the absence of TRP75. Bars, 10 µm. Download FIG S3, PDF file, 0.4 MB. Copyright © 2018 Luo et al. 2018 Luo et al. This content is distributed under the terms of the Creative Commons Attribution 4.0 International license.

### Impact of knockdown of TRP75-interacting proteins on E. chaffeensis infection.

**FIG 6:**
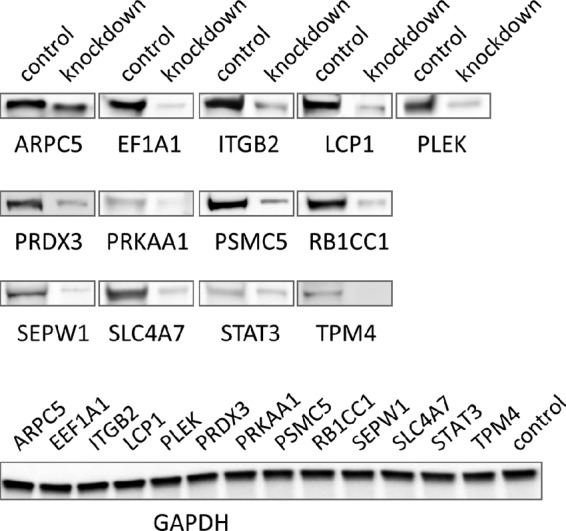
Verification of knockdown of TRP75-interacting host proteins by Western blotting. THP-1 cells were transfected with each specific or control siRNA, and at 2 days postinfection, Western blotting was performed to compare the amounts of protein. GAPDH was visualized as a control for the Western blot results.

The role of TRP75 in E. chaffeensis infection was further investigated by transfecting TRP75 target small interfering RNA (siRNA) into THP-1 cells and examining the impact on ehrlichial infection. At 2 days posttransfection, Western immunoblot results indicated that, compared with the unrelated control siRNA-transfected cells, the level of expression of all selected target proteins (*n* = 13) was substantially reduced in specific siRNA-transfected cells, demonstrating the successful knockdown of gene expression of TRP75 targets and the good efficiency of the siRNAs (Fig. 6). We did not observe apparent cell death after each knockdown.

**FIG 7:**
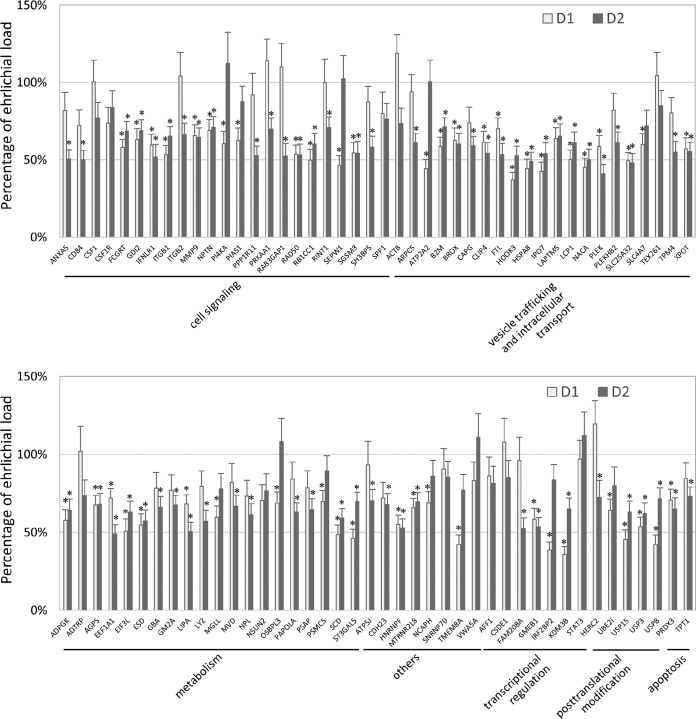
Influence of knockdown of TRP75-interacting proteins on *Ehrlichia* infection. THP-1 cells were transfected with target or control siRNA and were then infected by E. chaffeensis. The percentages of change in the levels of ehrlichial infection compared to the results seen with control scrambled siRNA-transfected cells were determined by qPCR at 1 and 2 days postinfection. E. chaffeensis *dsb* gene copy numbers were normalized to host *GAPDH* gene copy numbers. Data were from three independent experiments, and the values represent means ± standard deviations of the results (*, *P* < 0.05 [significantly different from control infection results]).

In total, 86 TRP75-interacting host proteins were knocked down by commercially available siRNAs, and the impact of each siRNA on ehrlichial load was detected by real-time quantitative PCR (qPCR) (Fig. 7). Overall, reduction of 74 (86%) TRP75 target proteins significantly inhibited ehrlichial infection, while only 12 (14%) had no significant impact on infection. Unlike the results seen in previous studies performed with TRP120, none of TRP75-interacting protein knockdowns was found to promote ehrlichial infection. At 1 day postinfection (p.i.), the knockdown of 50 (58%) proteins significantly inhibited ehrlichial infection, increasing to 63 (73%) at 2 days p.i. (Table 2). The results indicated that most of the identified TRP75-interacting proteins play a role in E. chaffeensis infection by promoting survival and that knockdown of all 13 target proteins selected for more-detailed study significantly reduced ehrlichial infection, except for STAT3, for which the result was likely related to the knockdown efficiency of STAT3 being lower than that of the other targets.

**TABLE 2:** Impact of TRP75-interacting protein knockdown on E. chaffeensis survival in host cells

Parameter	No. (%) of genes whose knockdown had an impact on E. chaffeensis infection			Total no. of genes
	Inhibition	Promotion	No significant change	
Day				
1	50 (58)	0	36 (42)	86
2	63 (73)	0	23 (27)	86
Overall	74 (86)	0	12 (14)	86
				
Category of primary function				
Cell signaling	20	0	3	23
Vesicle trafficking and intracellular transport	19	0	2	21
Metabolism	18	0	2	20
Others	6		2	8
Transcriptional regulation	4	0	3	7
Posttranscriptional modification	5	0	0	5
Apoptosis	2	0	0	2
Total	74	0	12	86

We further classified 86 TRP75 host targets, according to their primary functions that summarize major cellular processes directly related to host-*Ehrlichia* pathobiology, into the following seven categories: cell signaling, vesicle trafficking and intracellular transport, metabolism, transcriptional regulation, posttranslational modification, apoptosis, and others. Knockdown of most or all target proteins in each category had a significant negative impact on ehrlichial infection (Table 2). These results suggest that, similarly to other TRPs, E. chaffeensis TRP75 is a moonlighting effector that interacts with multiple host targets to regulate important host cellular processes.

## DISCUSSION

In recent years, we determined that ehrlichial TRP32, TRP47, and TRP120 are effector proteins that interact with numerous host proteins associated with various host cell processes (12, 13, 15). These findings have helped us to define the importance of bacterial effectors and their interactions with eukaryote hosts and to the understand complex mechanisms by which pathogens modulate the host cell to promote infection. In this study, another tandem repeat protein of E. chaffeensis, TRP75, was also revealed to be an ehrlichial protein that interacts with a diverse array of host proteins, suggesting that TRP75 is also a moonlighting protein and plays important roles in complex mechanisms to modulate host cell processes that support ehrlichial infection, consistent with other TRP effectors.

Selected TRP75 target proteins, including SLC4A7, ARPC5, LCP1, PLEK, TPM4, EEF1A1, ITGB2, PRDX3, PRKAA1, PSMC5, RB1CC1, SEPW1, and STAT3, were verified by coimmunoprecipitation and colocalization with TRP75 in THP-1 cells. Our previous Y2H results revealed that E. chaffeensis TRP32 and TRP120 interact with EEF1A1; therefore, the EEF1A1 protein is a converging interacting partner with ehrlichial TRPs (12, 13). The primary function of EEF1A1 is to help deliver aminoacyl tRNAs to the ribosome during protein translation; thus, it is highly conserved and extremely abundant and plays a very important role in eukaryotes (21). However, EEF1A is also a moonlighting eukaryotic protein with multiple functions, including transcription, enzyme regulation, autophagy, apoptosis, and cytoskeletal reorganization (22). The interactions of multiple TRPs with EEF1A1 presumably play different roles in ehrlichial survival strategies, but the association of TRPs with the common target protein implies the crosstalk of different cellular processes manipulated by multiple *Ehrlichia* effectors and perhaps more important roles of overlapping host cell targets.

Our Y2H data also identified some closely related or function-associated interacting partners of E. chaffeensis TRPs, including a member of solute carrier family, solute carrier family 4 member 7 (SLC4A7). We previously identified solute carrier family 2 member 3 (SLC2A3) interacting with TRP120, solute carrier family 25 member 42 (SLC25A42) and family 46 member 3 (SLC46A3) interacting with TRP47, and solute carrier family 43 member 3 (SLC43A3) interacting with TRP32 (12, 13, 23). The solute carrier (SLC) consists of a large series of membrane transport proteins, including 52 families and over 400 members identified so far. SLC family members are located in the membrane of cells and in other intracellular organelles and are the gatekeepers for all cells and organelles. SLCs transport highly diverse solutes from organic molecules to inorganic ions and maintain the balance of crucial components such as nucleotides, amino acids, carbohydrates, ions, and other chemicals (24). Therefore, SLCs play important roles in homeostatic control mechanisms, such as the regulation of the concentrations of oxygen, carbon dioxide, glucose, sodium, and potassium; of pH; of osmolality; and of temperature and many aspects of cellular physiology (25). SLC4A7 is a sodium bicarbonate cotransporter, while SLC2A3, SLC25A42, SLC46A3, and SLC43A3 transport glucose, coenzyme A and adenosine 3′,5′-diphosphate, catabolite, and nucleobase, respectively (26–29). Although the role and significance of solute carrier family members in ehrlichial infection of the macrophage are not clear, it is evident that TRPs interact with a large number of human solute carrier proteins to modulate host homeostasis and facilitate ehrlichial infection.

Among the TRP75-interacting proteins identified by Y2H, a group of actin-binding or actin-related proteins, including ARPC5, LCP1, PLEK, and TPM4, was of significant interest. Actins are highly conserved proteins that participate in many important cellular processes, including cell division and cytokinesis, cell signaling, cell/organelle/vesicle movement, and maintenance of the cytoskeleton (30). Inhibition of actin polymerization in E. chaffeensis-infected DH82 cells has been shown to prevent filopodium formation and passage of ehrlichiae from cell to cell (31). Binding of E. chaffeensis invasin EtpE to the cell surface protein DNaseX triggers the cytoskeletal rearrangement and filopodium formation required for bacterial entry (32). Thus, although the exact mechanisms of ehrlichial infection remain largely undefined, actins play important roles from ehrlichial entry into to exit from the host cell. These TRP75 target proteins have been found to be involved in actin nucleation, actin filament bundle assembly, and cytoskeleton organization; movement of the cell or of a subcellular component; phagocytosis; intracellular transport; and vesicle docking in exocytosis (33–36). We also reported previously that E. chaffeensis TRP47 interacts with another actin-binding protein, CAP1, and that TRP120 interacts with actin gamma 1 (ACTG1) and with actin-related protein 2/3 complex subunit 2 (ARPC2) (12, 15). Therefore, the associations of TRPs with actins, actin-binding proteins, and actin-related proteins suggest their importance in ehrlichial entry and exit.

Notably, we identified E. chaffeensis TRP75 as an interacting partner of many host apoptosis-related proteins, such as EEF1A1, ITGB2, PRDX3, PRKAA1, PSMC5, RB1CC1, SEPW1, and STAT3, although these proteins can also be classified into other categories of primary function. For example, PRDX3 is a mitochondrial protein with an antioxidant function which can negatively regulate apoptotic process, protect cells from oxidative stress, and promote cell survival in cancer (37); SEPW1 is a selenoprotein and an antioxidant involved in p53 signaling during cell apoptosis (38); STAT3 is in the STAT protein family, the members of which are key transcription activators involved in many cellular processes such as cell growth and apoptosis (39). We previously found that TRP32 interacts with a downstream target of p53, tumor protein p53 inducible protein 11 (TP53I11) (13), which is involved in the regulation of apoptosis (40). TRP120 interacts with intercellular adhesion molecule 3 (ICAM3) (12), which promotes drug resistance via inhibition of apoptosis, and with protein phosphatase 3 regulatory subunit B alpha (PPP3R1), which positively regulates TNF-related apoptosis (41, 42). Moreover, TRP32, TRP47, and TRP120 interact with a wide range of immunoglobulins and ribosomal proteins, such as IGL, IGKC, IGHA1, IGHV, IGLL1, IGLL5, RPL24, and RPL11 (12, 13, 23), which are associated with stabilization of antiapoptotic protein expression and p53-mediated apoptosis, respectively (43, 44). Most of TRP75 target proteins involved in apoptosis exhibited strong interactions with TRP75 by coimmunoprecipitation and immunofluorescence analysis. Therefore, TRPs may recruit different host apoptosis-related proteins to stabilize the cell or may induce programmed cell death by inhibiting or promoting apoptosis, respectively, to support ehrlichial replication and exit at different stages of infection.

Other TRP75 targets identified by Y2H include some host proteins involved in cell signaling, vesicle trafficking and intracellular transport, metabolism, transcriptional regulation, posttranslational modification, and other processes (see Table S1 in the supplemental material). Since our current and previous Y2H experience and RNA interference assay results indicated that the greater number of examined TRP-interacting proteins were confirmed to be genuine positive targets (12, 13, 15, 23), we anticipate that many of the host proteins identified in this study will be found to be involved in bona fide interactions with TRP75, which provides insight regarding its functions. Recently, we found that posttranslational modifications such as SUMOylation and ubiquitination of TRPs contribute to the function and diversity of interactions between TRP and host targets (9, 45–47). Many bacterial effectors are posttranslationally modified upon infection in order to regulate host processes and promote bacterial survival (48). Chlamydia trachomatis CPAF is also a moonlighting intracellular bacterium effector targeting multiple host and bacterial proteins to regulate host signaling pathways and promote infection (49, 50).

Our previous siRNA experiments revealed that knockdown of TRP120-interacting host proteins could either increase or decrease the levels of E. chaffeensis infection (23). In this study, most siRNAs of TRP75-interacting proteins reduced the bacterial load, indicating that these proteins are utilized by ehrlichiae to promote infection; however, no siRNAs of TRP75-interacting proteins increased the bacterial load, suggesting that TRP75 does not inhibit or degrade host target proteins as reported for TRP120 (47). siRNAs of other identified TRP75-interacting proteins (*n* = 12) were not found to have a significant impact on infection; that result could be related to the importance, interacting specificity, functional stage, or abundance of the target protein in *Ehrlichia* infection.

RNA interference assays indicated that inhibition of E. chaffeensis infection by knockdown of different targets could occur at different stages of infection, suggesting that these target proteins may interact with TRP75 at different times during E. chaffeensis infection. Some siRNAs had a significant impact on bacterial load at both 1 and 2 days p.i., indicating that these targets play roles at both the early and intermediate stages of infection. Knockdown of some TRP75 targets inhibited ehrlichial infection dramatically, implying more important roles in infection. Both the coimmunoprecipitation and immunofluorescence assays also indicated that TRP75 interacted more strongly with some target proteins, such as EEF1A1, SEPW1, and SLC4A7, also suggesting increased significance of these cellular interactions. In addition, the remarkable recruitment and redistribution of some host proteins in E. chaffeensis-infected cells further demonstrate where these interactions are occurring.

Future studies to understand the details of these novel TRP-host molecular interactions will help define the molecular mechanisms responsible for ehrlichial infection and survival.

## MATERIALS AND METHODS

### Cell culture and cultivation of E. chaffeensis.

Human monocytic leukemia cells (THP-1 [ATCC TIB202], from ATCC, Manassas, VA) were propagated as recommended by ATCC. E. chaffeensis (Arkansas strain) was cultivated in THP-1 cells as previously described (51).

### Antibodies and siRNAs.

The antibodies used in this study were rabbit anti-human EEF1A1, SLC4A7, and PCGF6 (Santa Cruz Biotechnology, Santa Cruz, CA); ITGB2, PRKAA1, PSMC5, and RB1CC1 (Cell Signaling Technology, Inc., Beverly, MA); GAPDH (glyceraldehyde-3-phosphate dehydrogenase), PLEK, and TPM4 (Proteintech, Rosemont, IL); SEPW1 (Sigma); mouse anti-ARPC5 and LCP1 (Santa Cruz); PRDX3 (Pierce, Rockford, IL); and STAT3 (Cell Signaling Technology, Inc.). All commercial antibodies used were tested and confirmed by the vendor using Western immunoblotting or immunofluorescent microscopy or both to ensure the specificity. The rabbit or mouse anti-TRP75 antibodies used have been described previously (9). All siRNAs used in this study were siGENOME SMARTpool siRNAs from GE Healthcare/Dharmacon (Lafayette, CO), which represent mixtures of 4 siRNAs and are guaranteed to silence target gene expression by at least 75%, providing advantages with respect to both potency and specificity. The negative-control siRNA was an siGENOME nontargeting siRNA pool.

### Yeast two-hybrid (Y2H) assay.

In order to identify protein-protein interactions between E. chaffeensis TRP75 and human cells, Y2H screening was performed by Hybrigenics Services (Paris, France) following a standardized procedure. Briefly, the coding sequence for TRP75 (amino acids 23 to 583) (GenBank accession number NC_007799.1 [region: 560279 to 562030]) was PCR amplified and cloned into vector pB27 as a C-terminal fusion to LexA. The construct was verified by sequencing the entire insertion before use as bait to screen a randomly primed human macrophage cDNA library constructed in pP6 (52, 53). More than 50 million clones (5-fold the complexity of the library) were screened using a mating approach with YHGX13 and L40ΔGal4 yeast strains as previously described (54). His^+^ colonies were selected, and the prey fragments of the positive clones were amplified and sequenced at their 5′ and 3′ junctions. The resulting sequences were used to identify the corresponding interacting proteins in the GenBank database (NCBI) using a fully automated procedure as previously described (55).

### Coimmunoprecipitation (Co-IP).

Co-IP of TRP75 from E. chaffeensis-infected THP-1 cells was performed using the specific antibody against selected TRP75-interacting target protein with a cross-link immunoprecipitation (IP) kit (Thermo Scientific/Pierce) according to the manufacturer’s instruction. Briefly, 10 µg of target protein antibody was bound to protein A/G Plus agarose and then covalently cross-linked by the use of a disuccinimidyl suberate cross-linker. E. chaffeensis-infected THP-1 cells (10^7^ at 3 days postinfection [p.i.]) were collected, washed with phosphate-buffered saline (PBS), and then resuspended in 1 ml of lysis/wash buffer that contained cOmplete Mini Protease Inhibitor Cocktail (Roche Diagnostics, Indianapolis, IN), 5 mM EDTA, and 1 mM phenylmethylsulfonyl fluoride (PMSF). The supernatant of cell lysates was collected, precleared using the control agarose resin with gentle mixing for 30 min at 4°C, and then incubated with the antibody-cross-linked resin with gentle mixing for 2 h at 4°C. The resin beads were washed three times with lysis/wash buffer and once with conditioning buffer before elution was performed with the elution buffer. TRP75 was detected by Western immunoblotting using rabbit anti-TRP75 antibody.

### Western immunoblotting.

Transfected THP-1 cell lysates were prepared using CytoBuster protein extraction reagent (Novagen/EMD, Gibbstown, NJ), supplemented with cOmplete Mini Protease Inhibitor Cocktail (Roche Diagnostics), 5 mM EDTA, and 1 mM PMSF. The Co-IP eluate or THP-1 cell lysate was separated by sodium dodecyl sulfate-polyacrylamide gel electrophoresis (SDS-PAGE), and then Western immunoblotting was performed as previously described (56), and the results were detected with a ChemiDoc-It2 515 imager (UVP, Inc., Upland, CA). Image acquisition was performed using VisionWorks software (UVP, Inc.).

### Immunofluorescence and confocal laser microscopy.

Uninfected or E. chaffeensis*-*infected THP-1 cells (3 days p.i.) were collected, and the indirect immunofluorescent antibody assay was performed as previously described (12). Confocal images were obtained with an LSM 510 Meta laser scanning confocal microscope (Zeiss, Germany) and LSM META software (version 4.0; Zeiss) and were analyzed with LSM Image Browser (version 4.2; Zeiss). Intensity correlation analysis was performed using Fiji ImageJ (version 1.51n; National Institutes of Health, MD) (57, 58). Intensity scatters exhibit the distribution of green and red pixels for the represented images. Positive PDM ([product of the differences from the mean] = [red intensity − mean red intensity] × [green intensity − mean green intensity]) values demonstrate different colocalization points for the represented images. The Manders colocalization coefficient (MCC) is represented on each PDM image next to the calculated region of interest demonstrating the strength of colocalization based on the intensity correlation.

### RNA interference.

RNA interference experiments were performed in THP-1 cells on a 96-well plate as previously described (23). Briefly, the cells were infected with cell-free E. chaffeensis at 1 day posttransfection of siRNA and were collected at 1 day and 2 days p.i. for Western blotting and quantitative PCR (qPCR) to determine knockdown levels and infection status, respectively. Since ehrlichiae matured into dense-cored cells at 3 days p.i. and some infected cells started to collapse and release ehrlichiae, samples were not collected at 3 days p.i.

### Quantification of E. chaffeensis.

Bacterial loads in THP-1 cell lysates were analyzed using real-time qPCR as previously described (23). Briefly, amplification of the integral ehrlichial disulfide bond formation protein (*dsb*) gene and amplification of the human glyceraldehyde-3-phosphate dehydrogenase (*GAPDH*) gene were performed separately, and gene copy levels were analyzed on the basis of the threshold cycle (2^−ΔΔ*CT*^) method. The percentage of change of ehrlichial *dsb* copy numbers relative to the control was normalized to detected levels of the host *GAPDH* gene.

### Statistics.

The statistical differences between experimental groups were assessed with the two-tailed Student’s *t* test, and significance was indicated by a *P* value of <0.05.
