# Supplementary material for: Ehrlichia chaffeensis TRP75 Interacts with Host Cell Targets Involved in Homeostasis, Cytoskeleton Organization, and Apoptosis Regulation To Promote Infection
Source: mSphere. 2018 Apr 11;3(2):e00147-18. doi: 10.1128/mSphere.00147-18 (PMC5909120; doi:10.1128/mSphere.00147-18)
Supplement: TABLE S1 [file sph002182515st1.pdf]

TABLE S1

No.	Gene symbol	Full name	Gene ID	Y2H hits
1	ACTB	actin, beta	60	3
2	ADPGK	ADP-dependent glucokinase	83440	1
3	ADTRP	androgen-dependent TFPI-regulating protein	84830	1
4	AFF1	AF4/FMR2 family member 1	4299	1
5	AGPS	alkylglycerone phosphate synthase	8540	2
6	ANXA5	annexin A5	308	1
7	ARPC5	actin related protein 2/3 complex subunit 5	10092	1
8	ATP2A2	ATPase sarcoplasmic/endoplasmic reticulum Ca2+ transporting 2	488	1
9	ATP5J	ATP synthase, H+ transporting, mitochondrial Fo complex subunit F6	522	1
10	B2M	beta-2-microglobulin	567	1
11	BROX	BRO1 domain and CAAX motif containing	148362	1
12	CAPG	capping actin protein, gelsolin-like	822	1
13	CD84	CD84 molecule	8832	1
14	CDH23	cadherin-related 23	64072	1
15	CLIP4	CAP-GLY domain containing linker protein family member 4	79745	1
16	CSDE1	cold shock domain containing E1	7812	1
17	CSF1	colony stimulating factor 1	1435	1
18	CSF1R	colony stimulating factor 1 receptor	1436	1
19	EEF1A1	eukaryotic translation elongation factor 1 alpha 1	1915	1
20	EIF3L	eukaryotic translation initiation factor 3 subunit L	51386	1
21	ESD	esterase D	2098	1
22	FAM208A	family with sequence similarity 208 member A (C3orf63)	23272	1
23	FCGRT	Fc fragment of IgG receptor and transporter	2217	1
24	FTL	ferritin, light polypeptide	2512	2
25	GBA	glucosylceramidase beta	2629	1
26	GDI2	GDP dissociation inhibitor 2	2665	2
27	GM2A	GM2 ganglioside activator	2760	1
28	GMEB1	glucocorticoid modulatory element binding protein 1	10691	1
29	HERC2	HECT and RLD domain containing E3 ubiquitin protein ligase 2	8924	1
30	HNRPF	heterogeneous nuclear ribonucleoprotein F	3185	1
31	HOOK3	hook microtubule-tethering protein 3	84376	1
32	HSPA8	heat shock protein family A (Hsp70) member 8	3312	1
33	IFNLR1	interferon lambda receptor 1	163702	1
34	IPO7	importin 7	10527	1
35	IRF2BP2	interferon regulatory factor 2 binding protein 2	359948	1
36	ITGB1	integrin subunit beta 1	3688	1
37	ITGB2	integrin subunit beta 2	3689	2
38	KDM3B	lysine demethylase 3B (jumonji domain containing 1B, JMJD1B)	51780	1

39	LAPTM5	lysosomal protein transmembrane 5	7805	1
40	LCP1	lymphocyte cytosolic protein 1 (L-plastin)	3936	2
41	LIPA	lipase A, lysosomal acid type	3988	1
42	LYZ	lysozyme	4069	2
43	MGLL	monoglyceride lipase	11343	2
44	MMP9	matrix metallopeptidase 9	4318	1
45	MT-CO1 (COX1)	mitochondrially encoded cytochrome c oxidase I	4512	1
46	MT-CO2 (COX2)	mitochondrially encoded cytochrome c oxidase II	4513	1
47	MT-ND2	mitochondrially encoded NADH dehydrogenase 2	4536	2
48	MTRNR2L8	MT-RNR2-like 8	100463486	4
49	MVD	mevalonate diphosphate decarboxylase	4597	1
50	NACA	nascent polypeptide-associated complex alpha subunit	4666	1
51	NCAPH	non-SMC condensin I complex subunit H	23397	1
52	NPL	N-acetylneuraminate pyruvate lyase (dihydrodipicolinate synthase)	80896	1
53	NPTN	neuroplastin	27020	2
54	NSUN2	NOP2/Sun domain family, member 2	54888	1
55	OSBPL3	oxysterol binding protein like 3	26031	1
56	PAPOLA	poly(A) polymerase alpha	10914	1
57	PDE4DIP	phosphodiesterase 4D interacting protein	9659	1
58	PI4KA	phosphatidylinositol 4-kinase, catalytic, alpha	5297	1
59	PIAS1	protein inhibitor of activated STAT 1	8554	1
60	PLEK	pleckstrin	5341	1
61	PLEKHB2	pleckstrin homology domain containing B2	55041	1
62	PPP1R11	protein phosphatase 1 regulatory inhibitor subunit 11	6992	1
63	PRDX3	peroxiredoxin 3	10935	2
64	PRKAA1	protein kinase AMP-activated catalytic subunit alpha 1	5562	1
65	PSAP	prosaposin	5660	1
66	PSMC5	proteasome 26S subunit, ATPase 5	5705	1
67	RAB3GAP1	RAB3 GTPase activating protein catalytic subunit 1	22930	1
68	RAD50	RAD50 double strand break repair protein	10111	1
69	RB1CC1	RB1-inducible coiled-coil 1	9821	1
70	RINT1	RAD50 interactor 1	60561	1
71	SCD	stearoyl-CoA desaturase (delta-9-desaturase)	6319	1
72	SEPW1	selenoprotein W, 1	6415	2
73	SGSM3	small G protein signaling modulator 3	27352	1
74	SH3BP5	SH3 domain binding protein 5	9467	1
75	SLC25A32	solute carrier family 25 member 32	81034	1
76	SLC4A7	solute carrier family 4 member 7	9497	1
77	SNRNP70	small nuclear ribonucleoprotein U1 subunit 70	6625	2
78	SPP1	secreted phosphoprotein 1	6696	1
79	ST3GAL5	ST3 beta-galactoside alpha-2,3-sialyltransferase 5	8869	1
80	STAT3	signal transducer and activator of transcription 3	6774	1
81	TEX261	testis expressed 261	113419	1

82	TMEM8	transmembrane protein 8A	58986	1
83	TPM4	tropomyosin 4	7171	1
84	TPT1(TCTP)	tumor protein, translationally-controlled 1	7178	1
85	UBE2I	ubiquitin conjugating enzyme E2 I	7329	2
86	USP15	ubiquitin specific peptidase 15	9958	1
87	USP3	ubiquitin specific peptidase 3	9960	1
88	USP8	ubiquitin specific peptidase 8	9101	1
89	VWA5A	von Willebrand factor A domain containing 5A	4013	1
90	XPOT	exportin for tRNA	11260	2
