# Supplementary figures and images for: Ehrlichia chaffeensis TRP75 Interacts with Host Cell Targets Involved in Homeostasis, Cytoskeleton Organization, and Apoptosis Regulation To Promote Infection
Source: mSphere. 2018 Apr 11;3(2):e00147-18. doi: 10.1128/mSphere.00147-18 (PMC5909120; doi:10.1128/mSphere.00147-18)

APRC5

TRP75

Merged

Scatter plot

PDM + MCC

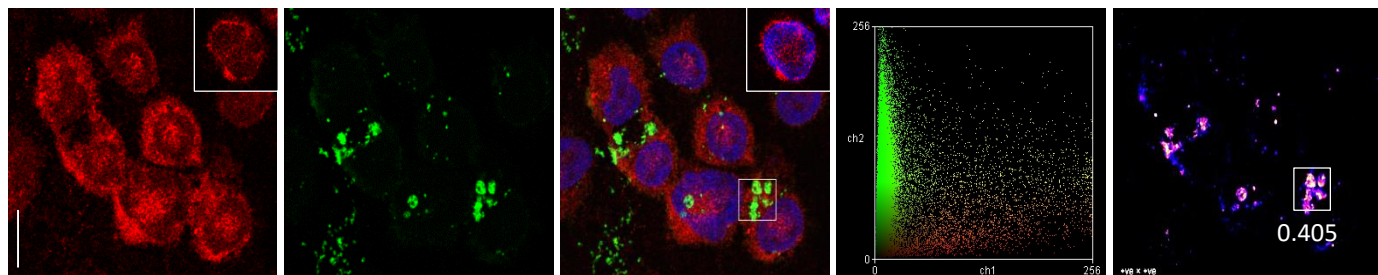

EEF1A1

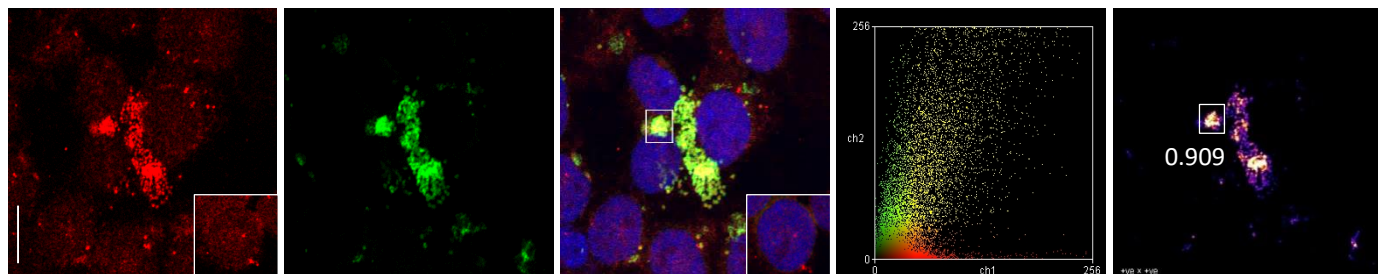

PLEK

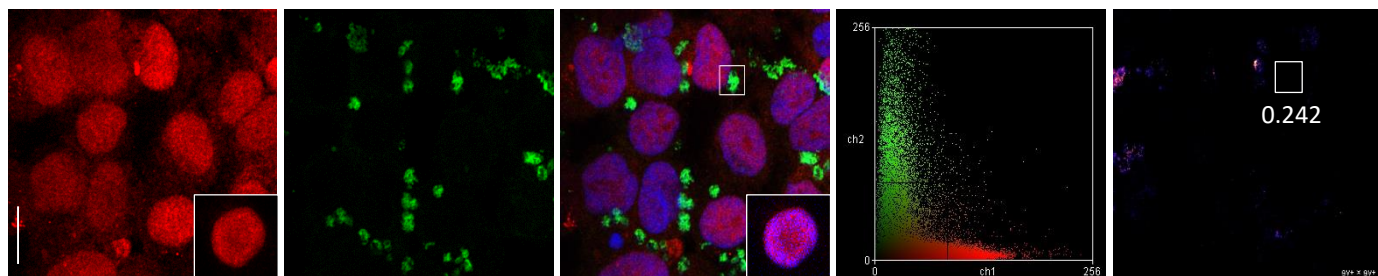

PSMC5

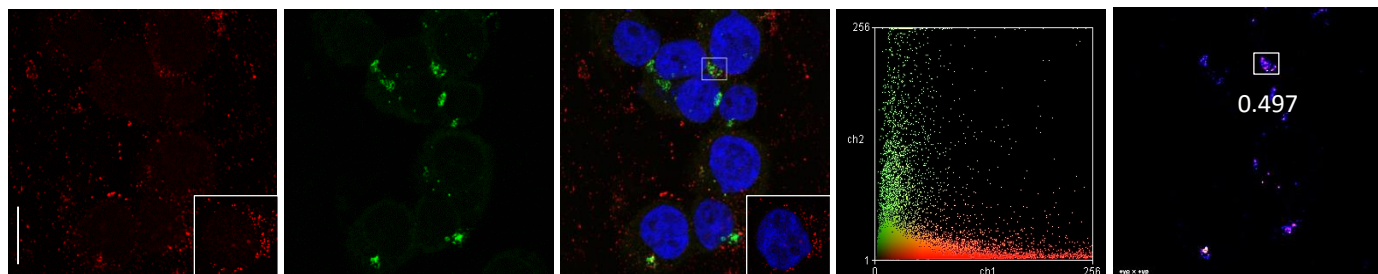

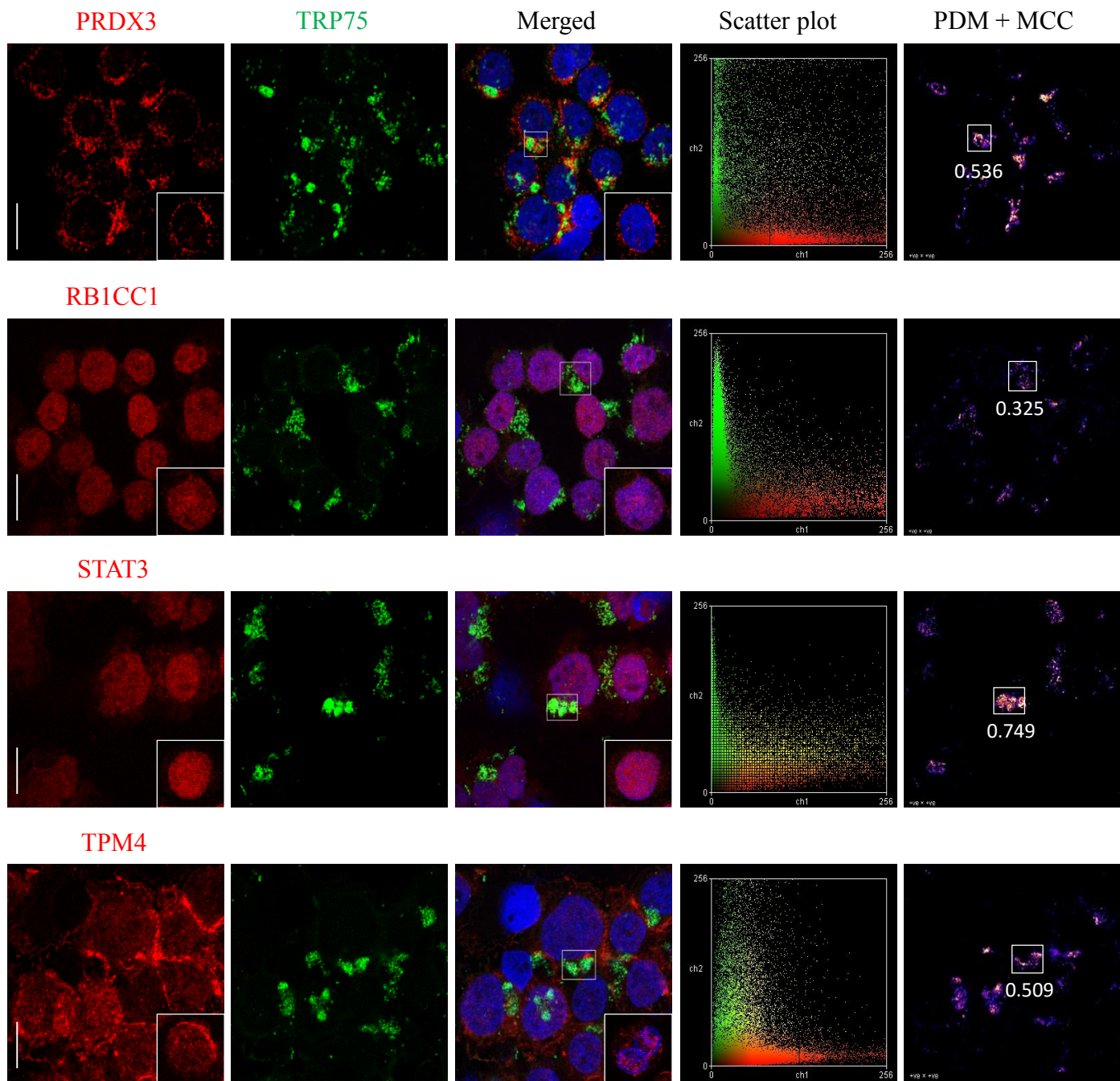

**FIG S1**

Supplement: FIG S1 [file sph002182515sf1.pdf]

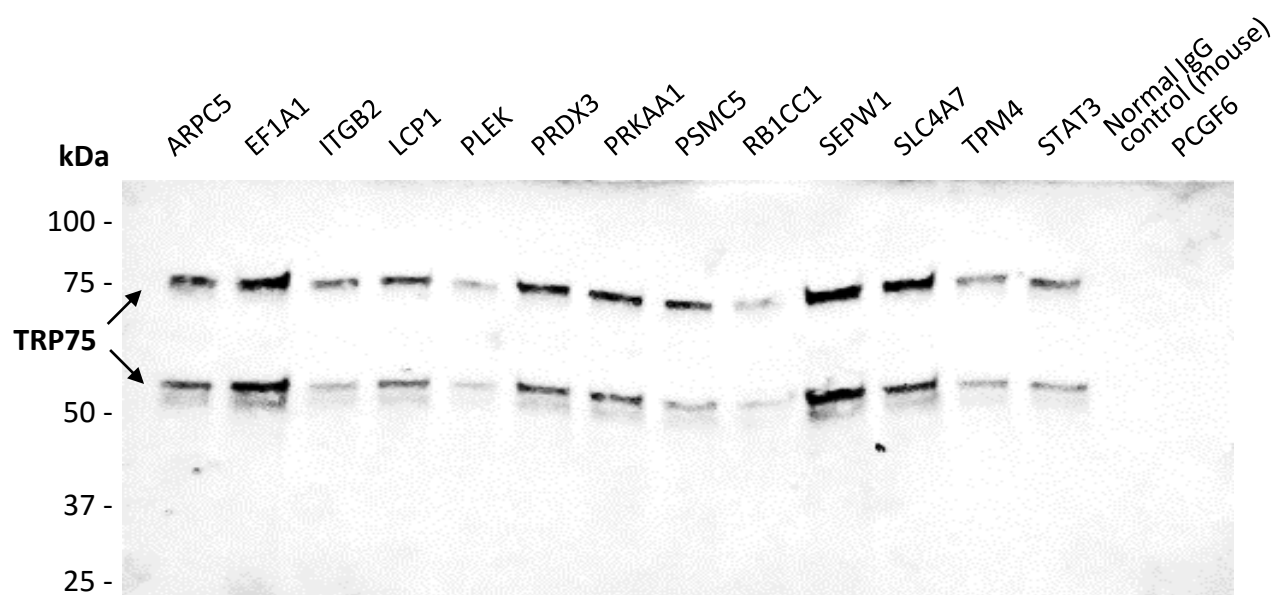

**FIG S2**

Supplement: FIG S2 [file sph002182515sf2.pdf]

ITGB2

TRP75

Merged

PLEK

TRP75

Merged

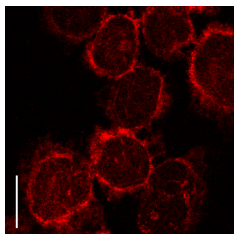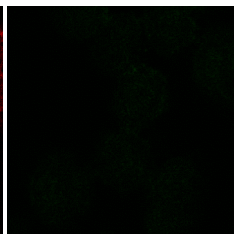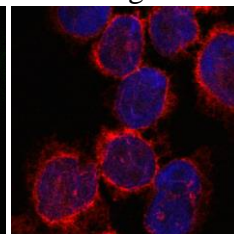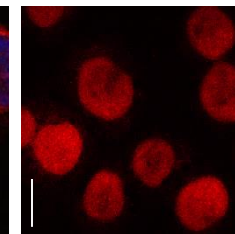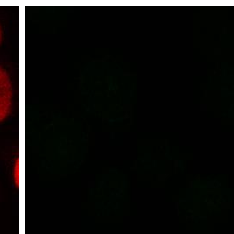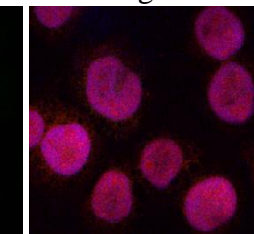

LCP1

PSMC5

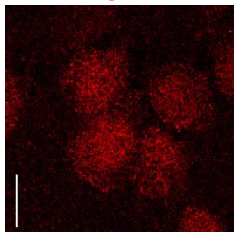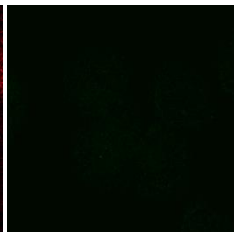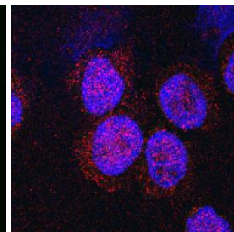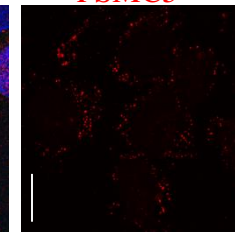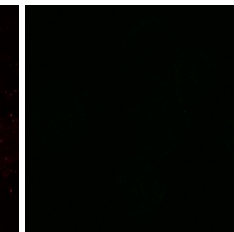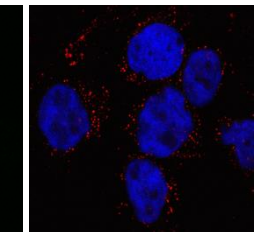

PRKAA1

PRDX3

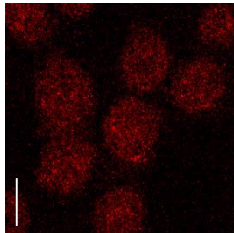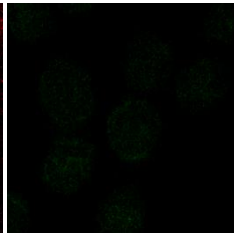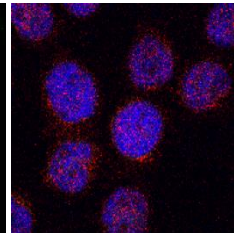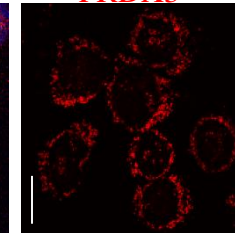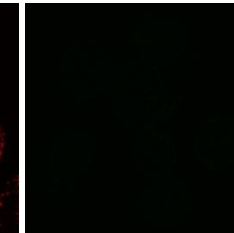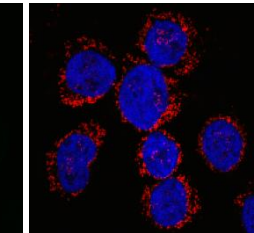

SLC4A7

RB1CC1

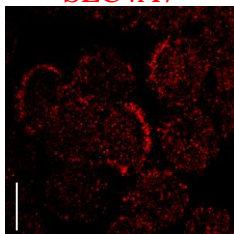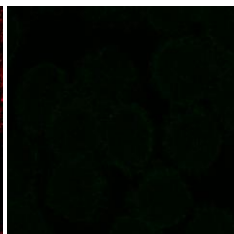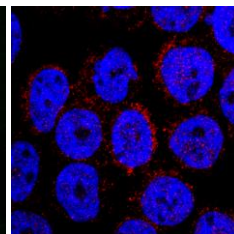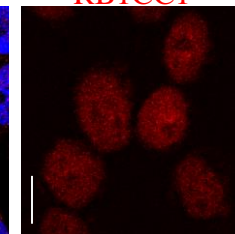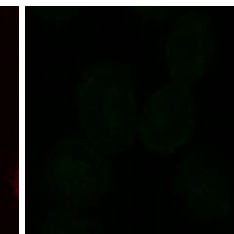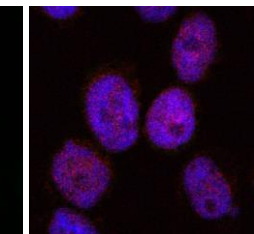

SEPW1

STAT3

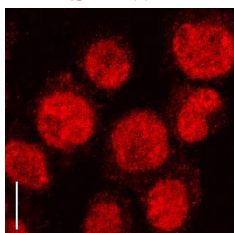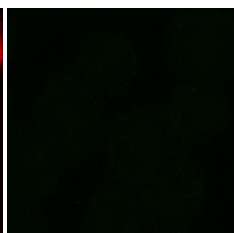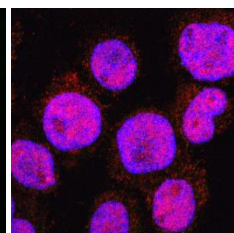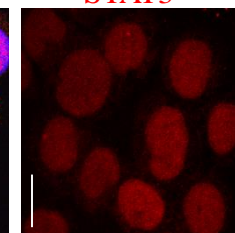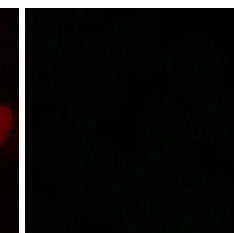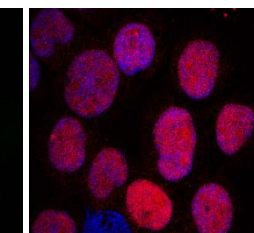

APRC5

TPM4

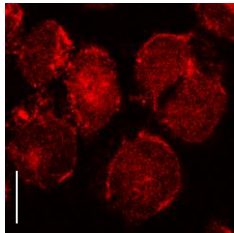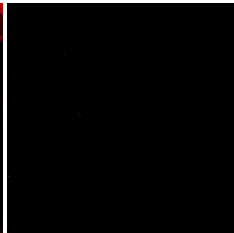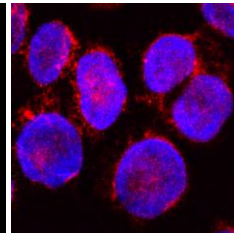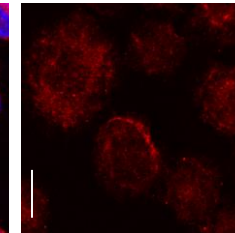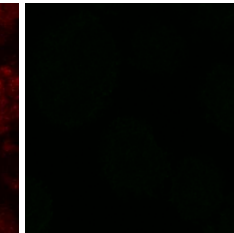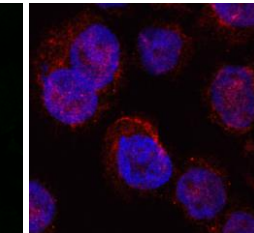

EEF1A1

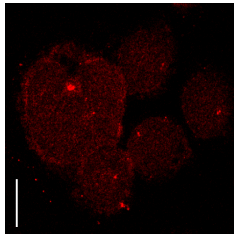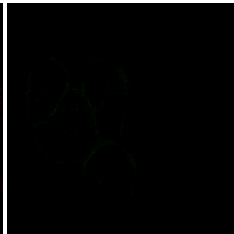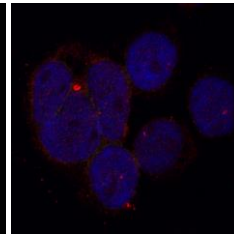

Supplement: FIG S3 [file sph002182515sf3.pdf]
